# Supplementary material for: Testing the association between blood type and COVID-19 infection, intubation, and death
Source: medRxiv. 2020 Sep 10:2020.04.08.20058073. Originally published 2020 Apr 11. Preprint. [Version 3] doi: 10.1101/2020.04.08.20058073 (PMC7276013; doi:10.1101/2020.04.08.20058073)
Supplement: 1 [file NIHPP2020.04.08.20058073-supplement-1.pdf]

medRxiv preprint doi: https://doi.org/10.1101/2020.04.08.20058073.this version posted September 10, 2020. The copyright holder for this preprint (which was not certified by peer review) is the author/funder, who has granted medRxiv a license to display the preprint in perpetuity.

It is made available under a CC-BY 4.0 International license .

Supplemental information

Supplementary Figure 1: ***Flow diagram of inclusion and exclusion criteria for the cohort used.*** *Numbers indicate the number of patients in each group. Groups on the right were excluded.*

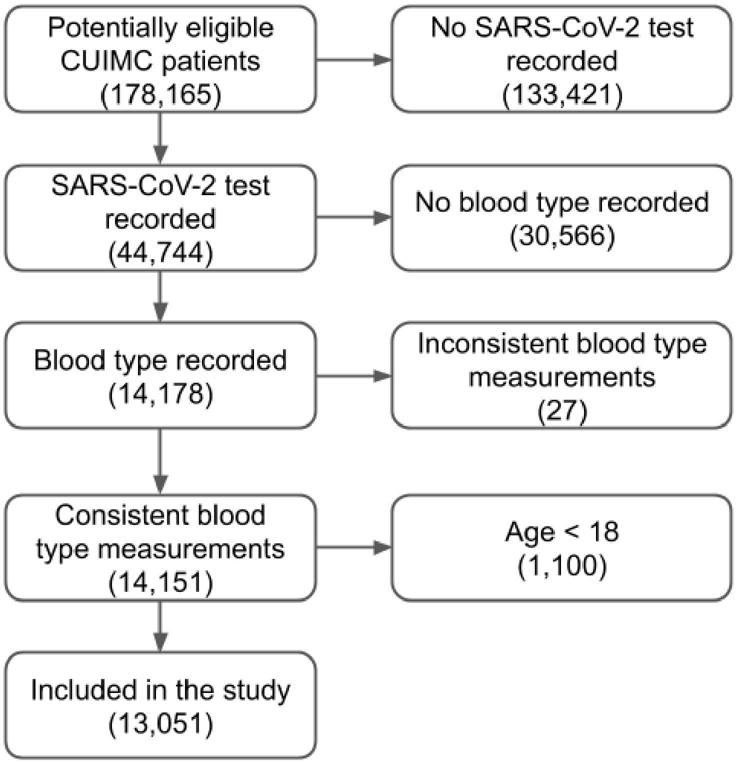

Supplementary Figure 2: ***Graphical model of the system under investigation.*** *We sought to estimate the total effects of blood type on the COVID-19 outcomes under investigation. Confounding can be controlled by adjusting for ancestry. Selection bias cannot be controlled fully, and as a result, our estimates are conditional on presentation to the hospital during the COVID-19 pandemic.*

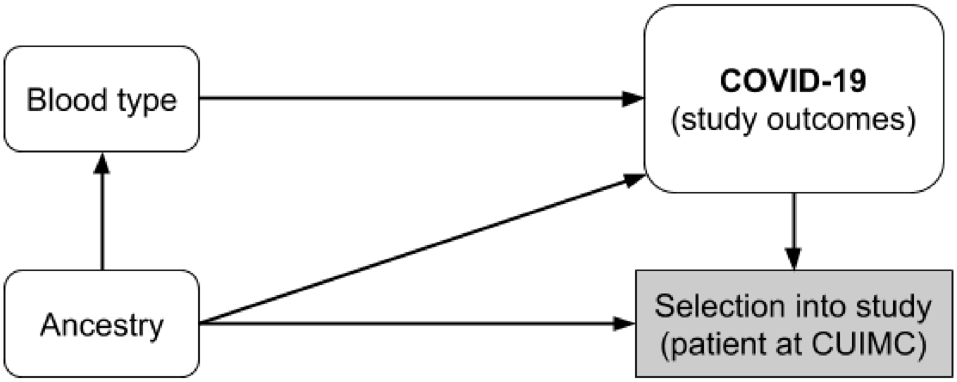

medRxiv preprint doi: https://doi.org/10.1101/2020.04.08.20058073.this version posted September 10, 2020. The copyright holder for this preprint (which was not certified by peer review) is the author/funder, who has granted medRxiv a license to display the preprint in perpetuity.

It is made available under a CC-BY 4.0 International license .

Supplementary Table 1: ***Chi-squared tests to evaluate whether the dependence between blood type and having received a test for SARS-CoV-2****. ABO had three degrees of freedom, while Rh(D) had one degree of freedom.*

Blood group	SARS-CoV-2 tested	non-SARS-CoV-2-tested	Chi-squared	p-value
ABO	A: 4298 (32.9%), AB: 559 (4.3%), B: 2033 (15.6%), O: 6161 (47.2%)	A: 34156 (32.7%), AB: 4405 (4.2%), B: 15590 (14.9%), O: 50305 (48.2%)	5.79	0.122
Rh(D)	neg: 1195 (9.2%), pos: 11856 (90.8%)	neg: 9644 (9.2%), pos: 94812 (90.8%)	0.0716	0.789
